# Dietary knowledge-attitude-practice status in hemodialysis patients: a latent profile analysis

**DOI:** 10.1186/s12889-024-18066-z

**Published:** 2024-03-18

**Authors:** Zhiqian Chen, Na Xu, Xinxin Chen, Xiaoyu Zhang, Shuqing Yin, Guanghui Xiao, Li Luo, Qun Liu, Chunyan Su

**Affiliations:** 1 Department of Nursing, Peking University Third Hospital, Beijing, China; 2 Department of Nephrology, Peking University Third Hospital Yanqing Hospital, Beijing, China; 3 Department of Nephrology, Peking University Third Hospital, Beijing, China; 4 Department of Nephrology, Fengtai Hospital of Integrated Traditional Chinese and Western Medicine, Beijing, China; 5 Department of Nephrology, Beijing Haidian Hospital, Beijing, China; 6 Department of Nephrology, Peking University International Hospital, Beijing, China; 7 Department of Nephrology, Fuxing Hospital, The Eighth Clinical Medical College, Capital Medical University, Beijing, China

**Keywords:** Hemodialysis patients, Dietary knowledge-attitude-practice, Latent profile analysis, Structural equation model, Influencing factors

## Abstract

**Background:**

Hemodialysis patients require a reasonable dietary intake to manage their disease progression effectively. However, there is limited research on these patients’ overall dietary knowledge, attitude, and practice (KAP) status. This study aimed to investigate the dietary KAP status and latent profiles in hemodialysis patients and identify sociodemographic and disease-related factors associated with these profiles and dietary practice.

**Methods:**

A multicenter cross-sectional study involving 425 hemodialysis patients was conducted. A dietary KAP questionnaire in hemodialysis patients was used to evaluate the dietary KAP of the patients. A structural equation model was employed to analyze the correlations between dietary knowledge, attitude, and practice. Multiple linear regression analysis was used to identify factors associated with dietary practice scores. Latent profile analysis was conducted to determine the latent profiles of dietary KAP, and binary logistic regression was used to explore the sociodemographic and disease-related characteristics associated with each KAP profile in hemodialysis patients.

**Results:**

The normalized average scores for dietary knowledge, attitude, and practice in hemodialysis patients were 0.58, 0.82, and 0.58, respectively. The structural equation model revealed significant positive correlations between dietary knowledge and attitude, and attitude and practice. Attitude played an indirect effect between knowledge and practice. Gender, cerebrovascular disease, and dietary attitude scores were identified as independent influencing factors for dietary practice scores. Two dietary KAP profiles were developed: a profile with general knowledge and attitude but low practice (40.2%) and a profile with general knowledge and attitude and high practice (59.8%). Binary logistic regression analysis indicated gender and monthly income per household significantly predicted membership in each KAP profile.

**Conclusions:**

The dietary practice of hemodialysis patients requires improvement. It is necessary to develop more individualized dietary interventions for these patients. Further exploration is needed to understand the motivation of patients to change their dietary behavior.

**Supplementary Information:**

The online version contains supplementary material available at 10.1186/s12889-024-18066-z.

## Background

Chronic kidney disease (CKD) is a significant global health problem, affecting approximately 14.3% of the population [1]. With its high prevalence, poor prognosis, and expensive treatment, CKD has become a significant concern [2]. As the disease progresses, CKD can lead to end-stage renal disease (ESRD) [3]. Hemodialysis (HD) is the most commonly used treatment for ESRD patients [4]. However, HD has limitations in terms of filtration and regulatory functions. Consequently, HD patients often experience a range of complications, including volume overload, hypertension, electrolyte disturbances, mineral and bone disorders, and malnutrition [5, 6]. These complications are closely related to the dietary intake of HD patients, which in turn affects disease control and mortality [7, 8]. Therefore, HD patients must adopt appropriate dietary practices to reduce complications and improve their prognosis.

The Knowledge-Attitude-Practice (KAP) theory is a widely recognized behavioral intervention theory that explains healthy behavior [9]. According to this theory, changing behavior involves two key steps: establishing beliefs and changing attitudes. The KAP theory has been extensively applied in developing questionnaires and investigating the KAP status in specific populations [10–12]. It has also been used in health education programs to prevent primary and chronic diseases [13–16]. Our research team has developed a dietary KAP questionnaire for HD patients based on the KAP theory. The questionnaire underwent validation through Delphi expert consultation and preliminary implementation in HD patients, demonstrating good reliability and validity [17]. However, the final version of the questionnaire has not been used to report the dietary KAP status in HD patients. Investigating the dietary KAP status of HD patients could help health professionals identify weaknesses in dietary management and develop corresponding interventions to improve patients’ dietary practice. Although a previous single-center survey investigated the KAP regarding hemodialysis and its complications in HD patients, it did not include dietary-related questions [18]. Therefore, more studies are needed to investigate the dietary KAP status of HD patients.

Latent profile analysis (LPA) is a person-centered approach using continuous variables to divide samples into meaningful subgroups based on similar characteristics [19]. It helps identify underlying characteristics of individuals based on their response patterns to specific topics, enabling a better understanding of individuals with different profiles [20, 21]. By employing LPA, we can explore the dietary KAP profiles of HD patients and identify sociodemographic and disease-related characteristics associated with different profiles. This approach will provide insights into the dietary KAP of HD patients.

Therefore, based on the KAP theory, this study aims to explore the latent profiles of dietary KAP and the corresponding sociodemographic and disease-related characteristics of each KAP profile in HD patients through LPA analysis. Additionally, we aim to investigate the dietary KAP status and the factors influencing dietary practices in HD patients. The findings of this study will provide essential evidence for healthcare professionals to develop targeted intervention strategies and improve the dietary KAP of HD patients.

## Methods

### Study design

This multicenter cross-sectional study employed convenience sampling to recruit 425 HD patients from five hospitals in Beijing, China, between June 1st, 2021, and July 31st, 2021.

### Participants

Participants informed consent was obtained from all participants. Eligible patients were 18 years or older, clinically diagnosed with ESRD, had undergone hemodialysis treatment for more than 3 months, had independent oral feeding without chewing and swallowing disorders, were conscious, and had unimpeded communication. Patients with acute comorbidities during the data collection period were excluded. It is approximated that there are 20–25 factors that affect dietary practice scores. Therefore, to avoid violating the principle that at least 10 cases are required for one variable in the multiple linear regression, a minimum sample size of 200–250 patients was needed [22]. The study received approval from the Medical Ethical Committee of Peking University Third Hospital (IRB2021–084-02).

### Data collection

#### Sociodemographic information

A self-designed questionnaire was used to collect sociodemographic data, including age, gender, marital status, education, occupational status, dialysis duration, payment method, primary disease, and comorbidities.

#### Dietary knowledge-attitude-practice questionnaire in hemodialysis patients

The questionnaire consisted of three sub-scales: dietary knowledge, attitude, and practice. The knowledge sub-scale comprised four dimensions: dietary potassium intake, protein energy intake, volume control, and dietary phosphorus intake. The attitude sub-scale was not dimensioned, while the practice sub-scale included four dimensions: dietary control, dietary monitoring, cooking skills, and selection of alternative condiments. The questionnaire contained 34 items, with 12 items in the knowledge sub-scale, 7 in the attitude sub-scale, and 15 in the practice sub-scale. Each knowledge item was scored as 0 or 1 (12 multiple-choice questions, with 1 point for a correct answer and 0 for a wrong answer); each attitude item was scored on a scale of 0 to 4 (0 for strongly disagree, 4 for strongly agree); and each practice item was scored on a scale of 0 to 4 (0 for never, 4 for always) as well. Higher scores indicated better dietary knowledge, attitude, and practice. The questionnaire demonstrated good content validity (0.79), with a Cronbach’s α coefficient of 0.868 and retest reliability of 0.845 [17]. The questionnaire is shown in the Appendix.

### Statistical analysis

SPSS25.0 was utilized for statistical description and analysis. Normally distributed continuous data were presented as the mean with standard deviation (SD), while non-normally distributed continuous variables were expressed using the median [interquartile range (IQR)]. Categorical data were presented as numbers (percentages). The two-tailed *t*-test and Mann-Whitney test were employed to compare two independent variables, while a one-way analysis of variance was used to compare multiple independent variables. The Bonferroni correlation was applied for post hoc tests. Categorical data were analyzed using the chi-square test. The correlation between continuous variables was examined using Spearman analysis. Multiple linear regression analysis was conducted to identify factors associated with dietary practice scores, and binary logistic regression analysis was performed to determine the independent influencing factors of KAP profiles. A two-tailed *P*-value < 0.05 was considered statistically significant.

Mplus 7.0 was employed to analyze latent profiles of dietary KAP in HD patients. The scores of 34 items of dietary KAP in HD patients were used as exogenous variables. Sequentially, 1 ~ 5 profiles were selected for analysis, and the fitting effect of the final model was evaluated using the following three indicators [23]. Firstly, the information evaluation index compared the difference between the expected value and the actual value with the Akaike Information Criterion (AIC), Bayesian Information Criterion (BIC), and adjusted Bayesian Information Criterion (aBIC) to determine the model fit. Smaller statistical values indicated a better fit. Secondly, the classification evaluation index assessed classification accuracy through the information entropy (Entropy), which ranges from 0 to 1. A value closer to 1 indicated a more accurate classification. Lastly, the likelihood ratio test compared the fitting differences between k-1 and k-category models using the Lo-Mendell-Rubin likelihood ratio test (LMR) and bootstrapped likelihood ratio test (BLRT). If *P* <  0.05 for LMR and BLRT, the k-category model is superior to the k-1 category model. While the above evaluation metrics provide a reference for profile decision-making, the interpretability of each category should also be considered when determining the best model.

AMOS 26.0 was utilized to test the mediating role of attitude between knowledge and practice. Bootstrapping was used to test the significance of the mediating effect. Non-significant and significant direct effects denoted fully mediated and partially mediated effects, respectively. We conducted 2000 bootstrap resamples and used 95% confidence intervals (CIs) to test for direct and indirect effects. The fit between the hypothesized model and the data was assessed by calculating absolute and incremental fit indices. A good model yields a minimum discrepancy divided by the degree of freedom (CMIN/df) <  3, a root mean square error of approximation (RMSEA) <  0.05 or at least 0.08, and goodness of fit index (GFI), adjusted goodness of fit index (AGFI), normed fit index (NFI), Tucker-Lewis index (TLI) and comparative fit index (CFI) >  0.90.

### Findings

#### Sociodemographic and disease-related information of hemodialysis patients

A total of 425 HD patients were recruited. 60.7% (258/425) patients were male, and 39.3% (167/425) were female, with age ranged from (23 ~ 97) years. See Table 1.

**Table 1:** The sociodemographic and disease-related information of HD patients

Items	n (%) / Median (IQR)	Items	n (%) / Median (IQR)
Age	60 (50, 68)	Occupation	
Marital status		Employee	191/424 (45.0%)
Married	364/425 (85.6%)	Self-employed	35/424 (8.0%)
No spouse^(a)^	61/425 (14.4%)	Farmer	79/424 (18.6%)
Education level		Unemployed	56/424 (13.2%)
Elementary school	63/423 (14.9%)	Others	63/424 (14.9%)
Junior high school	94/423 (22.2%)	Missing data	1
High school or secondary school	114/423 (27.0%)	Monthly income per household	
College	85/423 (20.1%)	< 1000 RMB	57/418 (13.6%)
Bachelor degree or above	67/423 (15.8%)	1000–2999 RMB	94/418 (22.5%)
Missing data	2	3000–4999 RMB	118/418 (28.2%)
Payment method		5000–9999 RMB	105/418 (25.1%)
Local medical insurance	314/422 (74.4%)	> 10,000 RMB	44/418 (10.5%)
Remote medical insurance	16/422 (3.8%)	Missing data	7
Urban and rural medical insurance	92/422 (21.8%)	Complication	
Missing data	3	Diabetes	148/425 (34.8%)
Primary disease		Hypertension	332/425 (78.1%)
Glomerulonephritis	75/425(17.6%)	Cardiovascular disease	82/425 (19.3%)
Hypertensive nephritis	102/425(24.0%)	Cerebrovascular disease	29/425 (6.8%)
Diabetic nephritis	110/425 (25.9%)	Malignant tumor	11/425 (2.6%)
Polycystic kidney	20/425 (4.7%)	Others	5/425 (1.2%)
Interstitial Nephritis	10/425 (2.4%)	Dialysis age (months)	80.00 (54.00, 125.75)
Others	108/425 (25.4%)	Duration per dialysis (hours)	4.00 (4.00, 4.00)
		Weekly frequency of dialysis	3.00 (3.00, 3.00)

*HD* Hemodialysis, *SD* Standard deviation, *IQR* Inter-quartile range

^(a)^No spouse: including unmarried, divorced, and widowed

### Dietary knowledge, attitude and practice status of hemodialysis patients

In the knowledge sub-scale, the mean scores per item in each dimension, in descending order, were “protein energy intake” “dietary phosphorus intake” “dietary potassium intake” and “volume control”. In the practice sub-scale, the mean scores per item in each dimension, in descending order, were “dietary control” “cooking skills” “selection of alternative condiments” and “dietary monitoring”. More details were shown in Table 2.

**Table 2:** Dietary knowledge, attitude and practice scores of HD patients

Dimensions	Mean (SD) / Median (IQR)	Standardized average score per item^(a)^
Dietary potassium intake	2.00 (1.00, 2.00)	0.58
Protein energy intake	1.00 (1.00, 2.00)	0.69
Volume control	2.00 (1.00, 3.00)	0.47
Dietary phosphorus intake	2.00 (2.00, 2.00)	0.66
Knowledge	7.00 (6.00, 8.00)	0.58
Attitude	21.5 (21.00, 28.00)	0.82
Dietary monitoring	10.00 (7.00, 13.00)	0.49
Dietary control	16.00 (13.00, 20.00)	0.66
Cooking skills	5.00 (3.00, 6.00)	0.58
Selection of alternative condiments	4.00 (3.00, 6.00)	0.55
Practice	34.68 (11.04)	0.58

*HD* Hemodialysis, *SD* Standard deviation, *IQR* Inter-quartile range

^(a)^Standardized average score per item = the average score of each dimension / the highest total score of each dimension

### Influencing factors of dietary practice scores in hemodialysis patients

#### Univariate factors influencing dietary practice scores in hemodialysis patients

The results showed that gender, marital status, monthly income per household, payment method, and cerebrovascular diseases were significantly correlated to the dietary practice scores (*P* <  0.05); dietary attitude scores were positively related to dietary knowledge scores (*r* = 0.265, *P* <  0.001), and dietary practice scores (*r* = 0.237, *P* <  0.001) in HD patients. See Table 3.

**Table 3:** Univariate factors influencing dietary practice scores in HD patients

	Mean (SD)	*t* / F	*P*
Gender		−3.109	0.002
Male	33.35 (10.52)		
Female	36.72 (11.54)		
Marital status		2.676	0.047
No spouse	33.89 (12.09)		
Married	34.81 (10.87)		
Monthly income per household		2.392	0.050
< 1000 RMB	32.32 (12.16)		
1000–2999 RMB	33.39 (11.03)		
3000–4999 RMB	34.18 (10.4)		
5000–9999 RMB	36.93 (10.37)		
> 10,000 RMB	36.18 (11.41)		
Payment method		3.362	0.036
Local medical insurance	35.41 (10.72)		
Remote medical insurance	35.5 (12.88)		
Urban and rural medical insurance	32.07 (11.42)		
Comorbidity - Cerebrovascular diseases		−2.061	0.040
No	35.27 (10.39)		
Yes	39.34 (8.17)		

*HD* Hemodialysis, *SD* Standard deviation

#### Independent influencing factors of dietary practice scores in hemodialysis patients

Using “male, no spouse, monthly income per household ≤ 1000 RMB, local medical insurance, and no cerebrovascular disease” as the reference group, the results showed that the gender (*β* = 0.233, *P* <  0.001), cerebrovascular disease (*β* = 0.154, *P* = 0.002), and dietary attitude scores (*β* = 0.146, *P* = 0.003) were the independent influencing factors of dietary practice scores, and the R^2^ = 0.079. See Table 4.

**Table 4:** Independent influencing factors of dietary practice scores in HD patients

	B	S.E.	*β*	*t*	*P*	95% C.I.
Gender	4.862	1.026	0.233	4.738	< 0.001	2.845, 6.880
Cerebrovascular disease	6.077	1.968	0.154	3.087	0.002	2.207, 9.947
Dietary attitude scores	0.320	0.108	0.146	2.968	0.003	0.108, 0.532

*HD* Hemodialysis

### The structural equation model of dietary knowledge, attitude, and practice in hemodialysis patients

**Fig. 1:**
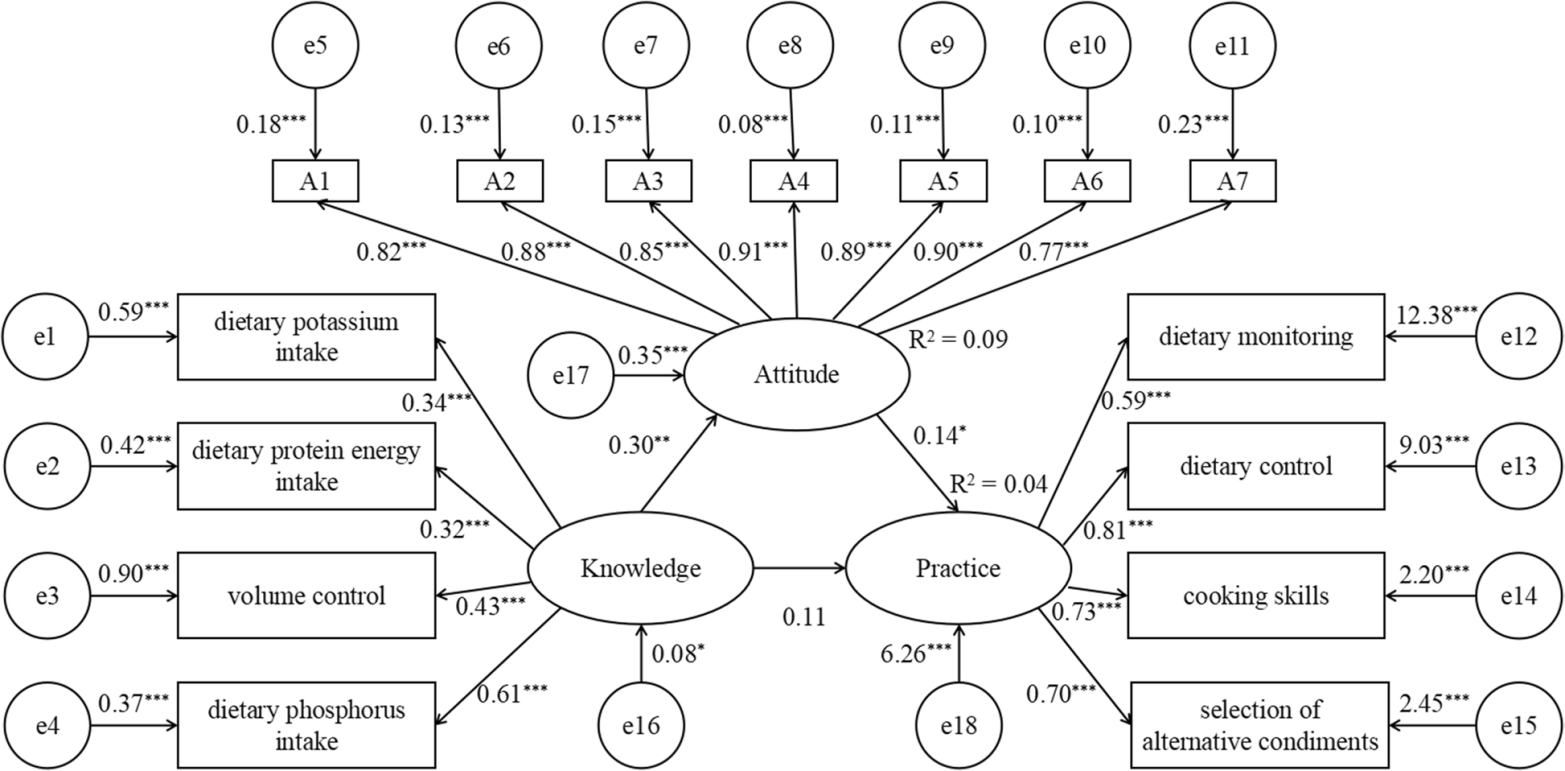
The structural equation model of dietary knowledge, attitude, and practice in hemodialysis patients. ^***^P < 0.001; ^**^P < 0.01; ^*^P < 0.05. Depiction: Rectangle shows observed variables, ellipses indicate potential variables, and circles represent residual terms

The structural equation model fit was ideal, with CMIN/df = 2.461, GFI = 0.937, AGFI = 0.913, TLI = 0.957, NFI = 0.941, CFI = 0.964, and RMSEA = 0.059 (Table 5). All models’ paths were statistically significant (*P* <  0.05), except for the knowledge-behavior path where *P* >  0.05. The R^2^ = 0.04. The structural equation model was shown in Fig. 1.

**Table 5:** Structural equation model of dietary knowledge, attitude, and practice in HD patients

	CMIN/df	GFI	AGFI	TLI	NFI	CFI	RMSEA
Evaluation Criteria	< 3 and > 1	> 0.8	> 0.8	> 0.9	> 0.9	> 0.9	< 0.08
Test results	2.461	0.937	0.913	0.957	0.941	0.964	0.059

*HD* Hemodialysis

### Profiles of dietary knowledge, attitude, and practice in hemodialysis patients

**Fig. 2:**
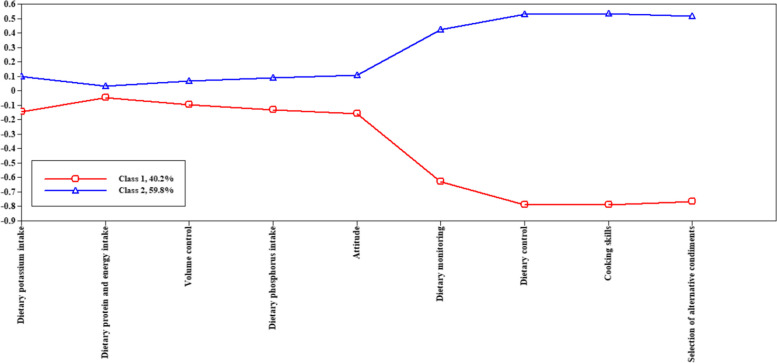
Description of the selected LPA profiles of the dietary knowledge-attitude-practice in hemodialysis patients. Depiction: Dietary knowledge, entries 1–4 in the horizontal coordinate; Dietary attitude, entry 5 in the horizontal coordinate; Dietary practice, entries 6–9 in the horizontal coordinate; Range of standardized scores, vertical coordinate; Class 1, general knowledge and attitude - high practice profile; Class 2, general knowledge and attitude - low practice profile

Normalize the scores for each dimension by using SPSS software. According to the LMR, the maximum number of classes to consider was two and BLRT was favorable each time to increase the number of classes. Nevertheless, the 2-class model had lower AIC, BIC, and aBIC values than the first. Furthermore, the 3-class model fitted better than the 2-class model. However, one category in the 3-class model had very low category probabilities (0.02). More detailed information can be found in Table 6. Considering the potential use of class membership as a variable for further analysis and the need for parsimony, the 2-class model was retained. Figure 2 illustrated the pattern of KAP scores for each identified profile. The two profiles, namely the “general knowledge and attitude - high practice profile” and the “general knowledge and attitude - low practice profile”, were primarily discriminated by different patterns of scores on the KAP dimensions.

**Table 6:** Fit indices for the latent profile analysis of dietary KAP in HD patients

Classes	AIC	BIC	aBIC	Entropy	LMR(p)	BLRT(p)	Categorical probability
1	10,881.868	10,954.806	10,897.685				1
2	10,498.116	10,611.574	10,522.720	0.754	< 0.001	< 0.001	0.40/0.60
3	10,383.079	10,537.058	10,416.471	0.838	0.055	< 0.001	0.57/0.41/0.02
4	10,265.507	10,460.007	10,307.686	0.797	0.042	< 0.001	0.02/0.19/0.52/0.27
5	10,140.777	10,375.798	10,191.743	0.879	0.107	< 0.001	0.19/0.39/0.02/0.13/0.26

*KAP* Knowledge-attitude-practice, *HD* Hemodialysis

### Factors influencing dietary knowledge, attitude, and practice profiles in hemodialysis patients

#### Univariate factors influencing dietary knowledge, attitude, and practice profiles in hemodialysis patients

The results showed that gender (*X*^*2*^ = 5.359, *P* = 0.021) and monthly income per household (*X*^*2*^ = 11.623, *P* = 0.020) were significantly associated with KAP profiles. More details were shown in Table 7.

**Table 7:** Univariate factors influencing dietary KAP profiles in HD patients

	General knowledge and attitude - low practice profile	General knowledge and attitude - high practice profile	*X*^*2*^	*P*
Gender			5.359	0.021
Male	114/258 (44.2%)	144/258 (55.8%)		
Female	55/167 (32.9%)	112/167 (67.1%)		
Monthly income per household			11.623	0.020
< 1000 RMB	27/57 (47.4%)	30/57 (52.6%)		
1000–2999 RMB	40/94 (42.6%)	54/94 (57.4%)		
3000–4999 RMB	56/118 (47.5%)	62/118 (52.5%)		
5000–9999 RMB	33/105 (31.4%)	72/105 (68.6%)		
> 10,000 RMB	11/44 (25.0%)	33/44 (75.0%)		

*KAP* Knowledge-attitude-practice, *HD* Hemodialysis

#### Independent influencing factors of dietary knowledge, attitude, and practice profiles in hemodialysis patients

The assignment of variables was shown in Table 8. The binary multivariate logistic regression results indicated that gender and monthly income per household were independent influencing factors of dietary KAP profiles in HD patients (*P* < 0.05). See Table 9.

**Table 8:** The assignment of variables

Variables	Assignment
KAP profiles	General knowledge and attitude - low practice profile = 0, General knowledge and attitude - high practice profile = 1
Gender	Male = 0, Female = 1
Monthly income per household	< 1000 RMB = (1, 0, 0, 0), 1000–2999 RMB = (0, 1, 0, 0), 3000–4999 RMB = (0, 0, 1, 0), 5000–9999 RMB = (0, 0, 0, 1), > 10,000 RMB = (0, 0, 0, 0)

**Table 9:** Independent influencing factors of dietary KAP profiles in HD patients

	B	S.E.	Wald	df	*P*	Exp(B)	95% C.I.
Gender	0.525	0.214	6.000	1	0.014	1.691	1.111, 2.574
Monthly income per household			12.649	4	0.013		
< 1000 RMB	−1.159	0.446	6.749	1	0.009	0.314	0.131, 0.752
1000–2999 RMB	−0.887	0.410	4.685	1	0.030	0.412	0.184, 0.885
3000–4999 RMB	−1.128	0.411	7.517	1	0.006	0.324	0.145, 0.725
5000–9999 RMB	−0.702	0.401	3.063	1	0.080	0.496	0.226, 1.088

*KAP* Knowledge-attitude-practice, *HD* Hemodialysis

## Discussion

The findings revealed that among the dietary KAP dimensions, HD patients had the highest score in the attitude dimension and the lowest in the knowledge and practice dimension. It suggested that while some patients had a positive attitude toward dietary management, they struggled to translate it into actual practice. It might be because dietary practice was related to various sociodemographic and disease-related factors in addition to dietary attitude (See Table 2). The linear regression model further indicated that female patients with cerebrovascular disease and better dietary attitudes exhibited better dietary practices, which aligned with previous studies [24, 25]. The possible explanation was that male patients often engaged in more social activities involving table culture, while women tended to be more health-conscious [24]. Additionally, dietary management played a crucial role in preventing and controlling cerebrovascular disease [26], and there were often common dietary risk factors for various diseases. Therefore, patients with complications might exhibit better dietary practices.

The results indicated that patients had the worst dietary knowledge of volume control. In terms of dietary practice, they had the worst management of dietary monitoring (mainly about monitoring water and salt intake and weight gain). The awareness, knowledge and skills related to volume control among HD patients still need further improvement. Other factors, such as comorbidities and poly-medication treatments, might also contribute to poor volume control [27]. Strict volume control was crucial for HD patients. Excessive water and salt intake could lead to volume overload, increasing the risk of high blood pressure, heart disease, and in severe cases, even left heart failure and death [28]. Therefore, it was essential to strengthen health education and interventions related to water and salt intake and volume control in HD patients in the future.

This study’s structural equation model revealed significant positive correlations between dietary knowledge and attitude, as well as attitude and practice. Moreover, the study found that attitude mediated between knowledge and practice, indicating that attitude influenced knowledge translation into behavior. These findings were consistent with the KAP theoretical model and previous findings [29, 30]. According to the KAP model, dietary behavioral changes in HD patients involved three successive processes: knowledge acquisition, belief generation, and behavior formation. Patients with sufficient dietary knowledge were more likely to develop a positive attitude toward dietary management and transfer to improved dietary behavior. Therefore, dietary education could promote positive dietary behavior among HD patients. While the study provided valuable insights, further exploration was needed to understand the specific mechanisms and factors involved in transforming dietary knowledge, attitude, and practice in HD patients.

The R^2^ value of the linear regression model was only 0.079, although higher than the R^2^ (0.04) of the structural equation model, suggesting that the KAP theory might have limitations in explaining dietary behavior change in hemodialysis patients. There were likely several other environmental and social factors that should have been considered in this study. However, previous studies demonstrated that the KAP theory could effectively explain behaviors such as influenza vaccine uptake among healthcare workers (R^2^ = 0.69) [29] and nutrition labeling among residents (R^2^ = 0.545) [31]. The disparity in results could be attributed to the dietary behavior being a relatively ingrained habit formed over a long period, influenced by various social, cultural, environmental, financial, and traditional factors [32]. Consequently, changing dietary behavior become more challenging. A previous study by Gao et al. [33] exploring the behavioral pathways explaining oral health disparity in children also revealed that patients’ health behavior was associated with not only knowledge and attitude but also ethnicity and socioeconomic status. Therefore, developing dietary interventions that consider individuals’ personalized characteristics, dietary knowledge and attitude was crucial. The KAP model could also be refined and revised to cater to specific behaviors by considering environmental, sociodemographic, and disease-related characteristics. New behavioral theory models could be developed to better explain and guide dietary behavior change.

This study identified two distinct profiles of dietary KAP patterns among HD patients: a low-practice profile and a high-practice profile. Interestingly, the study found that while the knowledge and attitude toward dietary intake were similar in both profiles, there was a significant difference in dietary practice. Changing patients’ knowledge and attitude might not change dietary behavior. Dietary practice was a complex behavior influenced by various personal and social factors [34–36]. Further analysis of the profiles revealed that the high practice profile consisted of more female patients with higher monthly household income, indicating that patients with higher socioeconomic status tended to have better dietary KAP. This could be attributed to their increased access to healthcare resources and higher expectations for quality of life [37–39]. Consequently, they were more likely to understand and adhere to health education and dietary management requirements, as well as had better conditions and resources to manage their dietary intake in daily life. In contrast, patients with lower socioeconomic status faced barriers to accessing healthcare resources. Studies found that higher prices deter city dwellers from making sustainable food choices, even when they were interested in sustainability [40]. Impoverished populations often struggled to afford nutrient-rich fresh foods, further exacerbating health disparities [41]. Efforts should be made to improve access to healthcare resources and promote health equity for patients with lower socioeconomic status, including increasing access to healthy foods.

### Study limitations

The participants were limited to HD patients in Beijing, China, and there might be variations in dietary KAP among patients from different geographical areas. In this regard, this study was designed to be a multicenter study conducted at different levels of hospitals to compensate for this shortcoming. Additionally, the cross-sectional study could not verify the causal relationship. Future multicenter, longitudinal clinical studies with large samples are needed to confirm these findings and causal relationships.

Furthermore, the linear regression and structural equation models explained only a small proportion of the variance in dietary practice among HD patients. Given the complexity of dietary behavior, future investigations should consider the roles of biological, environmental, policy, and other factors to gain a more comprehensive understanding of dietary behavior change. Additionally, combining qualitative and quantitative studies could provide insights into the motivations behind dietary behavior change in dialysis patients.

## Conclusion

This study found that HD patients’ dietary knowledge and attitude were better than their actual dietary practice. The study also identified dietary attitude as a positive mediator between dietary knowledge and practice. However, the R^2^ value of the structural equation model was small, indicating that other factors influence dietary practice. The study showed that dietary practice in HD patients is affected by dietary attitude, gender, and cerebrovascular diseases. The high practice profile consisted of more female patients with higher incomes. Therefore, Individualized interventions are needed to improve dietary practice, considering factors such as attitude, gender, and socioeconomic status. Future research should consider a broader population and explore additional factors influencing dietary behavior.

### Supplementary Information

**Supplementary material 1.**

## Data Availability

The datasets used during the current study are available from the corresponding author on reasonable request.

## Abbreviations

aBIC: Adjusted bayesian information criterion
AGFI: Adjusted goodness of fit index
AIC: Akaike information criterion
BIC: Bayesian information criterion
BLRT: Bootstrapped likelihood ratio test
CFI: Comparative fit index
CIs: Confidence intervals
CKD: Chronic kidney disease
CMIN/df: Discrepancy divided by the degree of freedom
ESRD: End-stage renal disease
GFI: Goodness of fit index
HD: Hemodialysis
IQR: Interquartile range
KAP: Knowledge-attitude-practice
LMR: Lo-Mendell-Rubin likelihood ratio test
LPA: Latent profile analysis
NFI: Normed fit index
RMSEA: Root mean square error of approximation
SD: Standard deviation
TLI: Tucker-Lewis index
