# Supplementary material for: Dietary knowledge-attitude-practice status in hemodialysis patients: a latent profile analysis
Source: BMC Public Health. 2024 Mar 18;24:836. doi: 10.1186/s12889-024-18066-z (PMC10946152; doi:10.1186/s12889-024-18066-z)
Supplement: Supplementary file 1 — Supplementary material 1. [file 12889_2024_18066_MOESM1_ESM.docx]

**Dietary Knowledge-Attitude-Practice Questionnaire in Hemodialysis Patients**

1. ***Dietary knowledge***

*Please select the appropriate one of the four options: A, B, C, and D, and fill in the line. Please do not leave items empty or missing.*

1. The primary sources of calories in the diet of hemodialysis patients are ____:
2. Fat, protein, water
3. Fat, protein, carbohydrate
4. Fat, carbohydrate, mineral
5. Fat, vitamin, mineral
6. Which of the following options is rich in high-quality protein?
7. Fat meat, egg yolk, beef, sea cucumber, fish
8. Milk, egg white, beef, fish, tofu
9. Vegetable, sweet potato, steamed bread, shrimp, crab
10. Tofu, bean sprout, soy milk, milk, egg
11. The daily salt intake of hemodialysis patients should not exceed ____:
12. 4 g
13. 6 g
14. 8 g
15. 10g
16. Which of the following options has a high salt content in food that hemodialysis patients need to restrict the intake?
17. Pickled food, sauces, ham sausage, fresh pepper
18. Pickled food, sauces, ham sausage, instant noodle
19. Pickled food, sauces, vinegar, instant noodle, sausage
20. Pickled food, sauces, apple, instant noodle, sausages
21. Which of the following options has a high water content in food that hemodialysis patients need to limit appropriately?
22. Water, steamed bread, watermelon, porridge, soup
23. Drink, fruit, vegetable, bread, porridge
24. Water, beverage, fruit, porridge, soup
25. Water, egg, vegetable, meat, soup
26. Weight gain between two hemodialysis sessions should be controlled at less than ____ of dry weight:
    A. 3%
27. 5%
28. 7%
29. 10%
30. The target of blood phosphorus control in hemodialysis patients is ____ mmol/L:
31. 0.86 - 1.35
32. 0.86 - 1.78
33. 0.86 - 1.90
34. 1.13 - 2.10
35. Which of the following options is high in phosphorus and should be minimized or avoided by hemodialysis patients?
36. Vegetables, fruits, egg yolks, steamed bread, beef
37. Rice, steamed bread, shrimp skin, sesame, fruit
38. Fresh milk, pork, eggs, peanuts, spinach
39. Egg yolk, cola, sesame sauce, mushrooms, dried small shrimps
40. The serum potassium of hemodialysis patients should be controlled within the range of ____ mmol/L:
41. 3.5 - 5.5
42. 3.0 - 5.0
43. 3.0 - 5.5
44. 3.5 - 6.5
45. Which of the following foods is high in potassium that hemodialysis patients need to limit or avoid eating?
    A. Banana, orange, peanut, melon seeds, dried fruit
46. Watermelon, apple, pear, spinach, tofu
47. Wax gourd, lotus root powder, soybean milk, cake, biscuit
48. Green pepper, onion, cucumber, rice, milk
49. Which of the following options does not contain high potassium that hemodialysis patients can choose to eat?
50. Bananas, oranges, peanuts, melon seeds, dried fruit
51. Watermelon, apple, pear, cucumber, wax gourd
52. Wax gourd, lotus root powder, soybean milk, potato, tomato
53. Green pepper, onion, cucumber, pork, milk
54. Which of the following cooking methods can effectively reduce potassium and phosphorus in the diet?
55. Cold salad
56. Boil in water, discard soup
57. Fried
58. Steaming
59. ***Dietary attitude***

*Please select the appropriate item in “Very disagree” “Disagree” “Uncertain” “Agree” and “Very agree” according to your situation and tick the box. Please do not leave the item empty or missing.*

|  | Very disagree | Disagree | Uncertain | Agree | Very agree |
| --- | --- | --- | --- | --- | --- |
| 1. Reasonable diet adjustment is significant for hemodialysis patients, which can prevent/improve hemodialysis-related complications. |  |  |  |  |  |
| 1. Reasonable energy and protein intake can prevent/improve malnutrition in hemodialysis patients. |  |  |  |  |  |
| 1. Strict water and salt intake restriction is beneficial to control weight gain during hemodialysis. |  |  |  |  |  |
| 1. Reasonable water and salt intake restriction can effectively control blood pressure and improve cardiac function. |  |  |  |  |  |
| 1. A low-phosphorus diet helps to control blood phosphorus and reduce the long-term complications of hemodialysis, such as cardiovascular disease and bone disease. |  |  |  |  |  |
| 1. A low-potassium diet helps maintain normal blood potassium levels and avoid myasthenia and cardiac arrest caused by hyperkalemia. |  |  |  |  |  |
| 1. Recording and checking your diet (3-day diet diary or 24-hour review) as recommended by your physician/dietitian can help you adjust your dietary intake. |  |  |  |  |  |

1. ***Dietary practice***

*Please select the appropriate item in “Always” “Often” “Sometimes” Rarely” and “Never” according to your diet in the last month and tick the box. Please do not leave items empty or missing.*

|  | Always | Often | Sometimes | Rarely | Never |
| --- | --- | --- | --- | --- | --- |
| 1. Regulate food types and intake according to protein and energy intake set by physicians/dietitians. |  |  |  |  |  |
| 1. Use a graduated water cup to calculate the water you drink daily. |  |  |  |  |  |
| 1. Control the intake of water-rich foods such as noodles, wontons, and soups, as well as vegetables and fruits with high water content, such as tomatoes and watermelons. |  |  |  |  |  |
| 1. Use a graduated salt spoon to calculate the daily salt intake. |  |  |  |  |  |
| 1. Control the intake of salt-rich foods such as pickled vegetables, salted fish, instant noodles, and ham sausages. |  |  |  |  |  |
| 1. Use soy sauce, sauce, etc., instead of salt for seasoning to reduce salt addition. |  |  |  |  |  |
| 1. Use seasonings such as fresh pepper and vinegar to reduce the amount of salt. |  |  |  |  |  |
| 1. Control dried fruits, dairy products, seafood, viscera, and other foods high phosphorus content. |  |  |  |  |  |
| 1. When cooking food with high phosphorus content, such as meat, cook it in boiling water first, remove the water and continue to cook. |  |  |  |  |  |
| 1. When limiting potassium intake, blanch the vegetables and remove the water before cooking high-potassium vegetables such as green leafy vegetables. |  |  |  |  |  |
| 1. Control the intake of foods high in potassium, such as dried fruits, fungus, mushrooms, fresh fruit juices, raw vegetables, and fruits high in potassium, such as oranges and bananas. |  |  |  |  |  |
| 1. Properly choose fruits and vegetables with low potassium content, such as papaya, apple, pear, hanging melon, etc., and pay attention to not excessive intake. |  |  |  |  |  |
| 1. Read food labels when buying processed food. |  |  |  |  |  |
| 1. Weigh yourself every morning to monitor weight gain. |  |  |  |  |  |
| 1. Conduct regular laboratory tests and pay attention to the test results to understand the effect of diet adjustment. |  |  |  |  |  |
